# (*RE*)Ba_2_Cu_3_O_7−*δ*_ and the Roeser-Huber Formula

**DOI:** 10.3390/ma14206068

**Published:** 2021-10-14

**Authors:** Anjela Koblischka-Veneva, Michael Rudolf Koblischka

**Affiliations:** 1 Experimental Physics, Saarland University, P.O. Box 151150, D-66041 Saarbrücken, Germany; anjela@shibaura-it.ac.jp; 2 Shibaura Research Laboratories, Shibaura Institute of Technology, 1-3-5 Toyosu, Koto-ku, Tokyo 135-8548, Japan

**Keywords:** superconductors, cuprates, transition temperature *T_c_*, Roeser–Huber formula, rare earths, oxygen distribution, flux pinning

## Abstract

We apply the Roeser–Huber formula to the (*RE*)Ba${}_{2}$Cu${}_{3}$O${}_{7-\delta }$ (*RE*BCO with *RE*= rare earths) high-$T_{c}$ superconducting material class to calculate the superconducting transition temperature, $T_{c}$, using the electronic configuration and the crystallographic data. In a former publication (H. P. Roeser et al., Acta Astronautica 2008, 62, 733–736), the basic idea was described and $T_{c}$ was successfully calculated for the YBa${}_{2}$Cu${}_{3}$O${}_{7-\delta }$ compound with two oxygen doping levels δ= 0.04 and 0.45, but several open questions remained. One of the problems remaining was the determination of $T_{c}$ for the δ= 0.45 sample, which can be explained regarding the various oxygen arrangements being possible within the copper-oxide plane. Having established this proper relation and using the various crystallographic data on the *RE*BCO system available in the literature, we show that the Roeser–Huber equation is capable to calculate the $T_{c}$ of the various *RE*BCO compounds and the effects of strain and pressure on $T_{c}$, when preparing thin film samples. Furthermore, the characteristic length, *x*, determined for the *RE*BCO systems sheds light on the size of the $\delta T_{c}$-pinning sites being responsible for additional flux pinning and the peak effect.

## 1. Introduction

Obtaining the superconducting transition temperature, $T_{c}$, for a given material by calculation requires commonly the use of band structure calculations [1,2,3,4,5,6,7], which are complicated and require considerable calculation power. In a series of papers, Roeser et al. have shown a different approach [8,9,10,11,12], where only the electronic configuration and a profound knowledge of the crystal structure, to define a characteristic length, *x*, are required to obtain $T_{c}$. The superconducting charge carrier wave is then in resonance with this length *x*, defining $T_{c}$. This approach was found to be valid for several cuprate high-$T_{c}$ superconductors (HTSc) as well for some iron-based superconductors (IBS), and with some specific modifications also for superconducting elements, as well as several metallic alloy superconductors, including NbTi, the A15-superconductors, and the metallic superconductor with the highest $T_{c}$, MgB${}_{2}$ [13].

Furthermore, this approach leads to a general relation between the characteristic length, *x*, and the inverse of $T_{c}$, which yields a linear relation with the slope $\frac{h^{2}}{\pi k_{B}}$. The resulting straight line follows the equation of a particle in a box [14] with the energy $h^{2}/\left(8M_{\mathrm{eff}}x^{2}\right)=\pi k_{B}T_{c}$ for n=1, where *n* is the number of Cu-O planes per chemical formula [12]. Thus, the so-called Roeser–Huber equation is a powerful relation to obtain information about $T_{c}$, as well as the related energy, *E* or Δ. So, the Roeser–Huber equation provides a link between the information of crystallographic databases and the respective electronic configuration to obtain valuable information about superconductivity. Although it is not the intention to replace band structure calculations by this approach, the simplicity of the calculation makes the Roeser–Huber equation to be an useful tool to address questions which are normally not answered in band structure calculations, and it might be especially useful to be incorporated into machine-learning based predictions of $T_{c}$ [15,16,17,18,19], or, for quickly checking existing predictions, with the linear relation as mentioned above.

For the YBa${}_{2}$Cu${}_{3}$O${}_{7-\delta }$ (abbreviated as YBCO or Y-123) cuprate HTSc compound, a successful calculation was presented in the first papers on this topic [8,9] for two oxygen doping levels, $\delta_{1}=$ 0.04 and $\delta_{2}=$ 0.45. In this case, the characteristic length *x* is related to the oxygen defect structure within the Cu-O-plane, which is the highway for superconductivity in this HTSc material. Although both calculations yielded proper data for $T_{c}$, it could not be explained why for the case of $\delta_{2}$ a different Cu-O-chain within the Cu-O-plane is responsible for superconductivity. Thus, we revisit here the calculation process on the base of several findings from the literature to give a proper foundation to the calculations. Having established this, we then proceed to variable oxygen content δ, the various rare-earth (*RE*)-based 123-type HTSc (abbreviated *RE*BCO), and the effects of strain and pressure on $T_{c}$. Furthermore, we show that the concept of minimal-size clusters with different oxygen content δ has direct impact on the $\delta T_{c}$-pinning [20], being responsible for the so-called fishtail anomaly or peak effect in magnetization loops of various HTSc materials [21,22,23,24].

## 2. Material and Model

### 2.1. *RE*BCO Unit Cell

**Figure 1:**
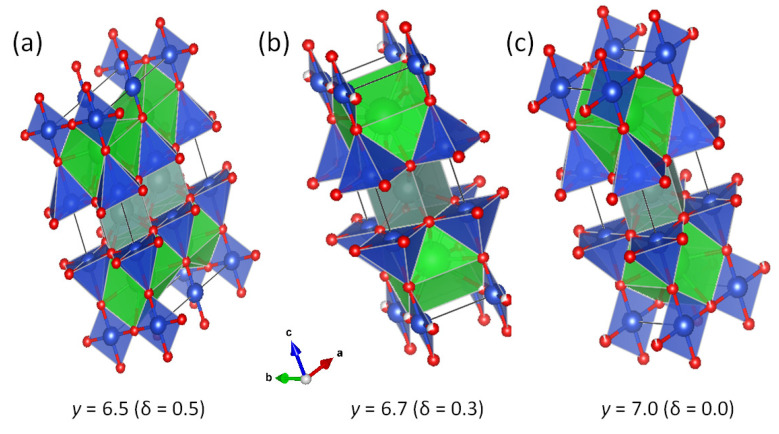
The crystal structures of (*RE*)BCO as a function of the oxygen content. (**a**) y= 6.5 (δ= 0.5), (**b**) y= 6.7 (δ= 0.3), and (**c**) y= 7 (δ= 0). Note that the doubled unit cell for δ= 0.5. The crystal structures were drawn using VESTA [25] and the cif-files from the ICDD database [27] and the Materials Project [28].

The unit cell and the superconducting properties of YBa${}_{2}$Cu${}_{3}$O${}_{7-\delta }$ depend strongly on the oxygen content, which can be given in the literature as oxygen deficiency, δ, oxygen content 6+x or y=7−δ. In Figure 1, we present three different orthorhombic YBCO unit cells with oxygen content varying between (a) y= 6.5 (δ= 0.5), (b) y= 6.7 (δ= 0.3), that is, underdoped YBCO, and (c) y= 7 (δ= 0), i.e., optimally doped YBCO, drawn using the VESTA software [25]. Overdoping of the unit cell is not possible in the YBCO system. The changes in the unit cells are due to the loading of oxygen into the structure. Note that the doubled crystal parameter for δ= 0.5. Below an oxygen content of δ= 0.5, YBCO is tetragonal and not superconducting. In case of all *RE*BCO materials, only one Cu-O-plane per unit cell contains the Cu${}^{3+}$-ions, which is considered to be the superconducting highway. The structural, thermal, mechanical, optical, and normal- and superconducting properties of the YBCO system were reviewed in Ref. [26].

### 2.2. The Roeser–Huber Approach

**Figure 2:**
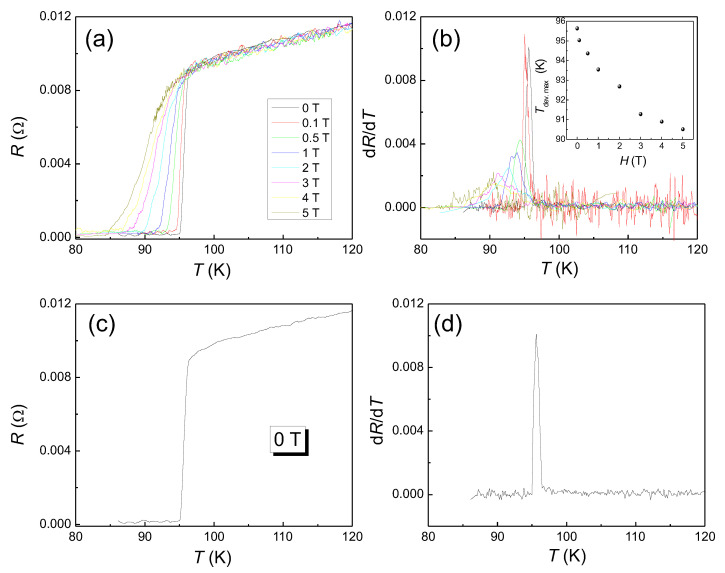
(**a**) Resistance vs. temperature of a melt-textured YBCO bulk prepared using the infiltration growth process in various applied magnetic fields. (**b**) The derivative dR/dT as function of *T*. The inset gives the maxima of dR/dT vs. the applied magnetic field. (**c**) The zero-field resistance curve and (**d**) dR/dT vs. *T*, illustrating the basic concept of the Roeser–Huber approach.

The Roeser–Huber approach interprets a resistance/resistivity vs. temperature curve as an integrated resonance curve. This idea is manifested regarding a typical resistance measurement of a melt-textured YBCO sample [29] fabricated using the infiltration-growth (IG) process [30]. Details about the resistance measurement technique can be found in Ref. [31]. In Figure 2a, the dependence R(T) is shown for various applied magnetic fields ranging between 0 T and 5 T. Figure 2b shows the corresponding derivative, dρ/dT, as function of temperature. The peaks obtained here shift towards lower temperatures on increasing magnetic field, as shown in the inset to Figure 2b. Taking now as an example the resistance at 0 T (Figure 2c) and its derivative (Figure 2d), the Roeser–Huber viewpoint of the resonance curve and its integral is becoming obvious.

Then, two times the characteristic length in the crystal structure, *x*, may be equal to the deBroglie wavelength of a Cooper pair ($\lambda_{\mathrm{cc}}$), so the crystal structure serves as a resonator to stimulate a coherent phase transition from a particle gas to a superconducting state. For HTSc, the charge carriers are Cooper pairs and their effective mass is equal to 2 times the electron mass, $m_{e}$. According to Refs. [8,9], the characteristic length, *x*, is related to the doping pattern within the Cu-O-plane containing the Cu${}^{3+}$-ions.

The main equation, i.e., the Roeser–Huber-equation, obtained for high-$T_{c}$ superconductors is

(1) $$\left[{\left(2x\right)}^{2}2M_{L}\right]n^{-2/3}\pi k_{B}T_{c}=h^{2}\;\;\;,$$

where *h* is the Planck constant, $k_{B}$ the Boltzmann constant, *x* the characteristic atomic distance, $T_{c}$ the superconducting transition temperature, $M_{L}$ the mass of the charge carriers, and *n* is a correction factor describing the number of Cu-O-planes in the unit cell. In the case of (*RE*)BCO materials, we have n= 1. For all the HTSc materials, the charge carrier mass is $M_{L}=2m_{e}$.

We may introduce an energy

(2) $$\Delta_{\left(0\right)}=\pi k_{B}T_{c}\;\;\;,$$

which can be considered as the pairing energy of the superconductor. Thus, we can write

(3) $${\left(2x\right)}^{2}\cdot 2M_{L}\Delta_{\left(0\right)}=h^{2}\;\;\;.$$

Using Equation (2) and regrouping, we arrive finally at

(4) $$\Delta_{\left(0\right)}=\frac{h^{2}}{2}\cdot \frac{1}{M_{L}}\frac{1}{{\left(2x\right)}^{2}}=\pi k_{B}T_{c}\;\;\;.$$

The energy $\Delta_{\left(0\right)}$ has of course a physical meaning and can thus be compared directly to the superconducting gaps as measured by photoemission spectroscopy (ARPES) [32,33] or tunneling (see, e.g., the review on scanning tunneling microscopy (STM) in Ref. [34]).

**Figure 3:**
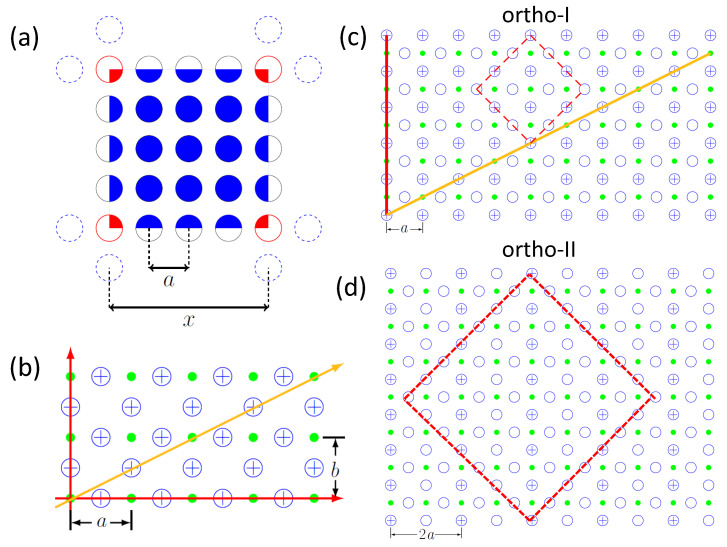
(**a**) Scheme for the determination of the characteristic length, *x*, for p= 5. The doping elements are indicated as blue, and the matrix elements in red. Each doping element at the corners of this cell are counting with only 1/4, and the other edge elements with 1/2. *a* is the distance between two matrix elements as indicated. (**b**) Schematic drawing of the Cu-O-plane following Ref. [9] with a=b. The oxygen atoms are drawn using ⨁ and the Cu atoms as •. The two Cu-O-Cu-O-chains identified there are indicated as red (▬) and orange (▬) vectors. *a* and *b* are the crystallographic axes of YBCO. Images (**c**,**d**) show the ortho-I (**c**) and ortho-II (**d**) structures redrawn from Poulsen et al. and Andersen et al. [35,36]. Oxygen vacancies are indicated using open blue circles (◯). Note the periodicity of 2*a* in (**d**). The red-dashed squares indicate the MSCs of the ortho-I and ortho-II phases. The orange and red lines identified by Roeser et al. [9] can be located here as well, as indicated in (**c**).

Now it is necessary to have a closer look how Roeser et al. [8,9] have found the characteristic length, *x*. In the first step, the given oxygen deficiency δ must be translated into a distance which can be described by the crystallographic parameters of the unit cell. Assuming a uniform doping density within the Cu-O-plane, Roeser et al. [9] employed the concept of an unit area for one doping element in a square planar configuration. In such a box, four doping elements (drawn in red) sit at each corner, the matrix elements are drawn in blue, and the box contains in total Σ elements. This situation is depicted in Figure 3a for p= 5 and Σ= 25. Each doping element at the corners of this cell is counting with only 1/4, and the other edge elements with 1/2. *a* is the distance between two matrix elements. In this way, the doping density can be calculated via

(5) $$\Sigma ={\left(p-2\right)}^{2}+\left(p-2\right)\cdot 4\cdot \frac{1}{2}+4\cdot \frac{1}{4}={\left(p-1\right)}^{2}\;\;\;.$$

Here, Σ denotes the number of elements in the box, and accordingly, the doping density is given by ${\left(\Sigma \right)}^{-1}$. *p* represents the number of elements within the unit area, and Σ and *p* are integers. The resulting doping distance (=average distance between the doping elements) is then given as x=(p−1)×a.

Particles participating in the superconducting state are moving collectively in phase and are equidistantly lined up on a straight line. Thus, in Ref. [9], two such Cu-O-Cu-O-chains were identified within the Cu-O-plane. In Figure 3b, this is shown as red and orange vectors. The green dots (•) denote the Cu atoms, whereas the blue crossed circles (⨁) denote oxygen atoms. In the case a=b, the vertical and horizontal red chains are equal to each other. The orange line is calculated as $\sqrt{{\left(2a\right)}^{2}+a^{2}}=\sqrt{5}a$.

In the case a≠b, the two red Cu-O-Cu-O-chains identified by Roeser et al. become unequal, as the vertical one is related to *b* and the horizontal one to *a*. Thus, the calculation of $T_{c}$ becomes more complicated as *a* will be always lower than *b*, defining a pair (a,b) with an orthorhombicity a/b. The orange, second Cu-O-Cu-O-chain is then calculated as $\sqrt{2a^{2}+b^{2}}$. This will be important for calculating $T_{c}$ of oxygen-deficient YBCO.

**Figure 4:**
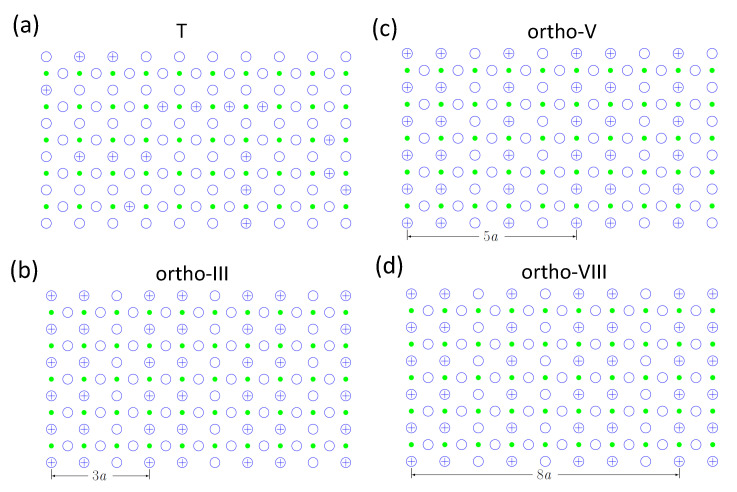
Idealized sketches of the phases (**a**) T, describing the tetragonal phase for δ> 0.7, (**b**) ortho-III (δ= 0.33) with a periodicity of 3a, (**c**) ortho-V (δ= 0.4) with a periodicity of 5a and (**d**) ortho-VIII (δ= 0.375) with a periodicity of 8a. Redrawn from Ref. [36].

Based on the identification of the Cu-O-Cu-O-chains which may play a role for the superconductivity, Roeser et al. calculated $T_{c}$ for two oxygen contents, $\delta_{1}=$ 0.04 (which corresponds to the optimum condition with the highest $T_{c}$) and $\delta_{2}=$ 0.45, which is the oxygen concentration of the plateau in the diagram when plotting $T_{c}$ as function of the oxygen stoichiometry (see Figure 4 below).

Employing the horizontal (red) Cu-O-Cu-O-chain for the calculation for $\delta_{1}=$ 0.04 worked out perfectly (see also Table 1). Here, it was calculated x=(p−1)×a=7×0.39nm, yielding a $T_{c}$ of 93 K for optimally doped YBCO.

In case of the lower oxygen doping, $\delta_{2}=$ 0.45, the situation was found to be different: Here, using the same horizontal Cu-O-Cu-O-chain as for the first case and p= 5, a resulting doping distance x=(p−1)×a=4×0.39nm=1.56 nm was found, which gives a $T_{c}$ being far too high (285 K). Then, the second (orange) Cu-O-Cu-O-chain was tried out yielding $x=1.56\;\mathrm{nm}\times \sqrt{5}=$ 3.49 nm. Using this value for *x*, $T_{c}$ can be calculated to be 57 K, which again fits perfectly well. The question left open in Ref. [9] was now why this second Cu-O-Cu-O-chain is more important for the lower $T_{c}$ value. This will be explained in the following section.

## 3. Results and Discussion

### 3.1. Explanation of the $T_{c}$ Variation in YBCO Using the Oxygen Clusters

To find an answer to this open question, a deeper look into the existing literature concerning the oxygen superstructures in the YBCO compound is required. Extensive research concerning the oxygen arrangement in the YBCO system was performed at Risø National Laboratory [39] in the 1990s employing neutron diffraction, synchrotron X-ray diffraction and simulation studies, and the main results were published in Refs. [35,36,39]. Using different measurement techniques and theoretical modeling, other groups [40,41,42] have also worked on the clarification of the oxygen structures in the Cu-O-planes.

Two idealized orthorhombic structures exist for the oxygen arrangement in the YBCO Cu-O-planes, the ortho-I (optimum doped, δ= 0, depicted in Figure 3c) and the ortho-II (δ= 0.5, depicted in Figure 3d) phases. Here, we have empty (◯) and filled (⨁) oxygen sites, and the Copper sites are symbolized by •. Comparing now the Roeser graph of Figure 3b with those in Figure 3c,d, one can identify the Cu-O-chains as investigated by Roeser et al. in the ortho-I and -II domains. The first straight, horizontal line of Roeser et al. does not exist at all in Figure 3c,d. This chain is here a chain of Cu-vacancy-Cu-vacancy, but its perpendicular counterpart does exist in the ortho-I phase, as well as in the ortho-II phase. However, in the ortho-II domain, every second Cu-O-Cu-O-chain is replaced by a Cu-vacancy-Cu-vacancy-chain, creating a new cell length of 2a, so there is an internal anisotropy of $T_{c}$ within the Cu-O-plane. In contrast, the second Cu-O-Cu-O-chain identified by Roeser et al. (drawn in orange), does exist in both ortho-I and ortho-II phases.

Using the asymmetric next nearest neighbor interaction (ASYNNNI) model [43], a minimal model was proposed by Poulsen et al. [35], which enabled the explanation of the $T_{c}$ variation with varying oxygen content. This model uses the assumption that only ordered oxygen domains or clusters exceeding a minimum size, dubbed minimal size clusters (MSC) can contribute to the charge transfer and influence the overall $T_{c}$ of the crystal. The ortho-I and ortho-II phases are the stable clusters of the 123-type material. In Figure 3b,c, the two different MSCs (4 × 4 for ortho-I and 8 × 8 for ortho-II) for the two phases are indicated as dashed-red squares. To calculate $T_{c}$ within this minimal model, the $T_{c}$ of a system with given oxygen content is obtained as a weighted average of the transition temperatures of the two types of ordered oxygen domains, if there are enough clusters considered.

An important finding of the ASYNNNI model simulations [35,36] is that there is a strong tendency of the oxygen vacancies within the superconducting Cu-O-planes to form chains, which lead to characteristic, mostly 1D-superstructures. Excess vacancies or excess oxygen atoms arrange themselves in chains, rather than being distributed randomly. The chain formation is a thermally activated process, and, in polarized in-situ reflection measurements using a YBa${}_{2}$Cu${}_{3}$O${}_{6.5}$ single crystal [44], information on the oxygen chain length could be obtained, such as the activation energy of 160 meV and the chain length as function of temperature, ranging between 4 and 40 nm. The lower value corresponds again nicely to the characteristic distance *x* of 3.49 nm determined by Roeser et al. [9]. A further consequence of this chain formation leads to interesting consequences, such as the paramagnetism of the Cu-O-chain fragments being formed by the oxygen doping [42,45]. This paramagnetism may be observed as an overlay to a normal magnetization curve if the overall magnetic moment is very small. In [45], a series of pure, sol-gel derived polycrystalline YBCO were analyzed, and a similar situation may be realized in YBCO nanofiber fabrics measured recently [46].

**Figure 5:**
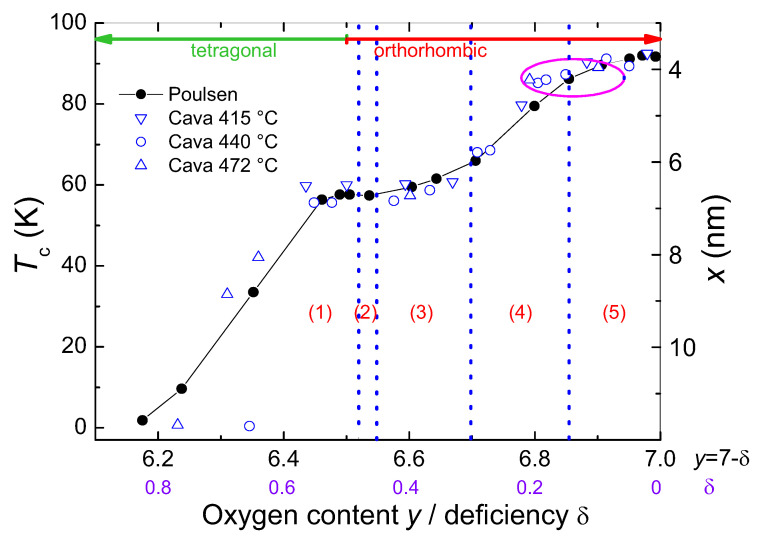
$T_{c}$ as function of the oxygen stoichiometry given as 7−δ or the oxygen deficiency δ. The open blue symbols are experimental data from Cava et al. [47,48], the filled black dots are simulation data of Poulsen et al. [35]. The line is a guide for the eye. Five regions labeled (1)–(5) could be identified (see main text). The magenta ellipse marks the region where the $\delta T_{c}$-pinning may take place. Figure redrawn from Ref. [35].

Thus, the oxygen arrangements in the Cu-O-plane can now be illustrated using idealized schemes at some specific oxygen contents. The pure tetragonal phase (labeled T, δ> 0.7) is illustrated in Figure 4a, where an alignment of finite length chain fragments can be observed. The ortho-I and ortho-II phases were already introduced in Figure 3c,d. Later refined research came up with more orthorhombic phases extending the schemes, namely ortho-III (δ= 0.33), ortho-V (δ= 0.4), and ortho-VIII (δ= 0.375) [36] as illustrated in Figure 4b–d, which have sequences of full and empty rows of oxygen with cell lengths of 3*a*, 5*a*, and 8*a*. Only the T and ortho-I phases establish a long range order, and the ortho-III, -V, and -VIII phases are essentially of 2D character, in contrast to ortho-II with 3D character. Following the phase diagram established in Ref. [36], we only need to consider the ortho-I and ortho-II clusters to describe $T_{c}$ above δ= 0.5, and the chain formation of the T-phase gives an idea how to interpret the large values of *x* obtained in Figure 5. Islam et al. [40] concluded finally that an ortho-IV phase with 4*a* modulation is obtained close to the optimal doping at x= 6.92. This implies that the oxygen superstructure consists of are clusters which extend typically over 3–6 unit cells in the *a,b*-plane and less than 1 unit cell along the *c*-axis. If we compare this value (δ= 0.08/6 unit cells) with the result of Roeser et al. (δ= 0.04/7 unit cells), this comes strikingly close.

The superconducting transition temperature (and also the transition width) of YBCO strongly depends on the oxygen stoichiometry, as shown in Figure 5. Many experimental data were obtained in the first years after the discovery of the YBCO compound [47,48,49,50,51,52,53,54]. Figure 5 combines experimental data from Cava et al. [47,48] with the simulations of Poulsen et al. [35], showing the good agreement achieved using the ASYNNNI model.

The computer simulations made in Ref. [35] showed that the degree of structural disorder and lateral heterogeneity of oxygen order varies considerably as a function of the oxygen content y=7−δ (oxygen deficiency δ) for fixed temperature. Below y=6.3 (δ= 0.7), we have the pure tetragonal phase T. For 0.7≥δ≥0.5 (stage (1)), the ortho-II phase is diluted by vacancies. At the plateau (0.5 ≥δ≥ 0.47, stage (2)), the pure ortho-II phase is realized, and for 0.47 ≥δ≥ 0.35 (stage (3)) the ortho-II phase prevails, but with defect structures of the ortho-I phase. In the range 0.35 ≥δ≥ 0.15 (stage (4)), the ortho-I phase dominates with inclusions of defect structures of the ortho-II phase, and for 0.15 ≥δ>0 (stage (5)), the ortho-I phase is diluted by vacancies. These five stages are indicated in Figure 5, labeled (1)–(5).

Poulsen et al. wrote in their paper “Analysis of the size dependence shows that the stoichiometry at the onset of superconductivity, and thereby the form of $T_{c}\left(y\right)$ up to the 58-K plateau, is dependent on the size of the ortho-II MSCs. On the other hand, the $T_{c}\left(y\right)$ variation between the 58-K and 93-K plateaus requires the presence of 4 × 4 MSCs but is, to some extent, robust to an increase of their size” [35]. This implies that the size of the ortho-II clusters plays an important role in region (1), but the size of the ortho-I clusters is not important in the regions (4) and (5); or in other words, the ortho-I clusters show their high-$T_{c}$ behavior in all possible sizes. In contrast, to have a strong $\delta T_{c}$-pinning, we need a region, where the Cu-O-plane is dominated by the ortho-I clusters, and disconnected ortho-II clusters with the minimum size can provide the $\delta T_{c}$-pinning sites embedded within a strongly superconducting matrix. This situation is realized in the magneta marked area of Figure 4.

A consequence of the $T_{c}$ calculation by Poulsen et al. [35] is that the calculation of the two doping levels at $\delta_{1}=$ 0.04 and $\delta_{2}=$ 0.45 as done by Roeser et al. [8,9] is fully sufficient to calculate $T_{c}$ of a YBCO sample for all oxygen stoichiometries: We just need to calculate a weighted average of the two different $T_{c}$’s to obtain the proper $T_{c}\left(y\right)$. Here we give one example: If we assume a total of 1000 clusters of an optimally doped sample, then the replacement of 50 clusters with ortho-II clusters will reduce $T_{c}$ from 93 K to 91.2 K, which fits to the situation of Ref. [37]. On the other hand, we can recalculate *x* for each configuration *y*, resulting in an averaged value for the characteristic length, *x*. Thus, it is possible to obtain a value for *x* corresponding to each $T_{c}$. This is illustrated on the right axis in Figure 4. As a larger *x* implies a lower $T_{c}$, the largest *x* is obtained for the lowest $T_{c}$, so x= 26.35 nm for $T_{c}=$ 2 K and 11.78 nm for 10 K. These large values for *x* describe the distance between the ortho-II clusters, not the size of the clusters. This situation changes when δ= 0.5 is reached: Now, x= 3.49 nm, which may correspond to the ortho-II cluster size as we will see in Section 3.2. According to this, the 4 × 4 clusters of the ortho-I phase have a minimal size of 2.7 nm.

To summarize this section, we can identify the Cu-O-Cu-O-chain used by Roeser et al. [8,9] to calculate $T_{c}$ for the optimum doped YBCO as the perpendicular chain (‖a as drawn in red in Figure 3b,c) in the ortho-I cluster. This chain does exist many times in the crystal, so consequently, it will dominate $T_{c}$. In *b*-direction, this chain does not exist, which implies that only the *a*-axis is important for $T_{c}$. For the oxygen deficient case $\delta_{2}=$ 0.45, there are two cases playing a role: The first one described by the red chain in the vertical direction, with its number reduced to 50%, and the second one in the horizontal direction due to the characteristic pattern defined by the chains and vacancies with a period of 2a. Then, calculating the vectorial addition of the two directions in the case a=b, we obtain $\sqrt{(a^{2}+{\left(2a\right)}^{2}}=\sqrt{5}a$, which corresponds to the orange chain as described by Roeser et al. Thus, we could clarify the role of the second chain in Roeser’s calculations: the chain itself is not important, it is the vectorial addition of the two directions within the ortho-II phase.

### 3.2. Discussion of the $\delta T_{c}$-Pinning and the Minimal Size Clusters

The peak effect or fishtail effect in the magnetization loops of *RE*BCO may have two types of origins, (i) due to the spatial variation of the oxygen content or (ii) by a variation of $T_{c}$ due to doping with other metallic dopants or the *RE*-Ba-solid solution in case of the light-*RE* elements, which can substitute for Ba [55].

Firstly, we have a look at the oxygen-induced fishtail effect, which is the most puzzling one as it can be made vanishing upon oxygen loading the sample [21]. In Figure 5, the magenta ellipse between the regions (4) and (5) marks the area where the $\delta T_{c}$-pinning can be active. It is essential that we have a strong superconducting matrix (ortho-I structure), where clusters of the ortho-II structure are embedded. These clusters with a lower $T_{c}$ can be driven normal by applying an external magnetic field. To be effective as flux pinning sites, the ortho-II clusters should not be too large or being interconnected with other ones, and, thus, the region in Figure 5 for observing the fishtail effect is limited. Thus, the discussion of internal granularity [56] is fully justified, only that the grain or cluster size is in the nanometer range. The values determined for *x* give us now a clue how large the ortho-II clusters must be to provide an effective $\delta T_{c}$-pinning. The ortho-II clusters must have in the minimum case an extension of $x=\lambda_{cc}/2$, where $\lambda_{cc}$ denotes the wavelength of the charge carrier material wave. Thus, $x_{\min}=$ 3.49 nm as calculated above. Here, we must note that *x* is larger than 2ξ as measured from resistivity data ($\xi_{ab}\left(0\right)=$ 1.41 ± 0.04 nm [57]). The strong superconducting matrix around the ortho-II clusters may introduce superconductivity to the clusters, so we must add 2 ×ξ/2 to $x_{\min}$ to ensure that the entire ortho-II cluster shows the lower $T_{c}$ value. Thus, a minimum cluster size of 4.5–5 nm results to provide strong $\delta T_{c}$-pinning.

Creating the $\delta T_{c}$-pinning by doping metal ions into the Cu-O-plane or changing $T_{c}$ by the RE/Ba solid solution creates much stronger $\delta T_{c}$-pinning which cannot be removed by oxygen annealing. As an example, in Ref. [39], the doping effect by Co-ions embedded within the Cu-O-plane was discussed. As consequence, the plateau in the $T_{c}$ vs. *y*-diagram (Figure 5) is altered to a shoulder for fixed Co content as the Co-ions attract oxygen.

Scanning tunneling microscopy (STM) imaging of the superconducting Cu-O-plane does not work out properly for YBCO and its derivatives. Low temperature cleaving splits the YBCO crystals between the Ba-O- and the Cu-O-chain-layers [58,59]. In the more recent Ref. [60], LDOS mapping was employed to visualize details of this Cu-O-chain layer, and a 1D-modulation with a length of 1.3 nm was found, indicating a proximity-coupled superconductivity in the Cu-O-chain layers. Nevertheless, this demonstrates how important oxygen vacancies and their arrangements can be for the superconductivity of the YBCO compound. In other HTSc materials, especially the Bi${}_{2}$Sr${}_{2}$CaCu${}_{2}$O${}_{8+\delta }$ (Bi-2212) compound, many imaging experiments of the Cu-O-planes were successfully performed [61,62,63,64]. A very important result was that the spatial variation of $T_{c}$ could be visualized in form of so-called gap maps showing the spatial variation of the measured superconducting gaps, defined as the distance between the first peaks above (below) the Fermi level, which is not necessarily representing a superconducting gap [62]. These observations in the Bi-2212 compound are surely also important for the Cu-O-plane in the (*RE*)BCO compounds.

### 3.3. (RE)BCO Compounds

**Figure 6:**
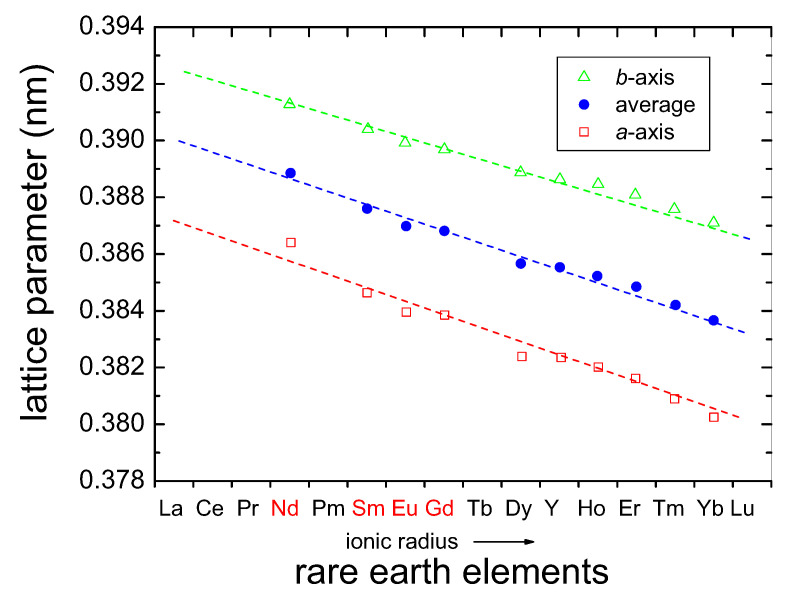
Cell parameters of various *RE*BCO compounds, taken from the ICDD database redrawn from Ref. [38]. The order of the elements on the horizontal axis follows the ionic radius of the *RE*-elements. The important light rare earth elements (LRE) are marked in red. Data are shown for the *a*-axes (□) and the *b*-axes (▵), as well as their average size (•).

Replacing the Y-atom in the 123 structure by other rare-earth materials leads to a change in the crystal parameters, as the other RE-atoms have different ionic radius as compared to Y. The cell parameters for the possible *RE*BCO compounds were investigated in Ref. [38] for thin films. Figure 6 presents their results. Here, the rare earth elements are given in the order of their ionic radii [65,66], and it leads to a linear decrease in the lattice parameters (a,b) as a function of the ionic radius. The *a*-axis is always larger than the *b*-axis, and the plot indicates also the average of *a* and *b*. The most commonly used substrates for the production of thin film materials, SrTiO${}_{3}$ (STO, a= 0.3905 nm) and LaAlO${}_{3}$ (LAO, a= 0.379 nm), show only a small misfit to the cell parameters of *RE*BCO, but it does produce some stress/strain to the *RE*BCO material.

Table 1 presents in the first part the calculated $T_{c}$-values for the two compositions of YBCO according to Ref. [9] with a=b= 0.390 nm. The resulting $T_{c}$’s are 93 K for optimum doping and 57 K for the plateau. If we use the optimum condition obtained in Ref. [37] with a=b= 0.385 nm and δ= 0.04, we find a $T_{c}$ of 95.6 K and for δ= 0.45, a value of $T_{c}=$ 58.55 K. This is clearly higher than any other compound in the list, so we must assume that δ of the YBCO coated conductor samples of Ref. [37] is higher. The formalism presented in Section 2.2 enables to calculate back the oxygen content using the $T_{c}$-data of Ref. [37]. Here, we find δ= 0.10, which is a reasonable value for coated conductor sample.

Several *RE*BCO compounds were also calculated for δ= 0.04 and using the literature data collected in Ref. [38]. Here, it is important to note that the calculated $T_{c}$ increases with decreasing *x* (increasing *RE* ionic radius). The values for $\Delta_{\left(0\right)}$ may be compared to the results of STM/STS-measurements. In Ref. [67], STM/STS was performed on NdBCO single crystals, where a peak-to-peak distance of 56 ± 4 meV for the optimally doped sample with a measured $T_{c}$ of 95 K was found, 70.6 ± 1.5 meV for a slightly underdoped sample with $T_{c}=$ 93 K and 117 ± 25 meV for a heavily underdoped sample with $T_{c}=$ 76 K. The data of the optimally doped sample come very close to the calculated $2\Delta_{\left(0\right)}$, whereas the underdoped samples show an increasing peak-to-peak distance, in contrast to $\Delta_{\left(0\right)}$ decreasing. However, as already mentioned before, the peak-to-peak distance does not necessarily correspond to the superconducting gap [62].

**Figure 7:**
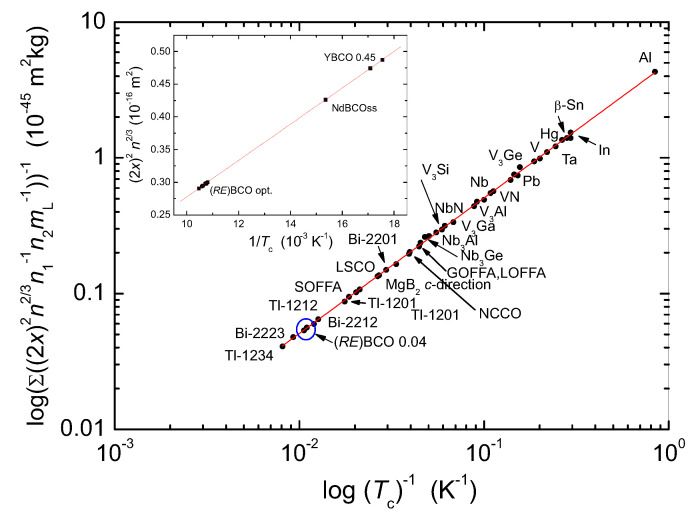
Roeser–Huber plot for various superconductors. The Roeser–Huber line (▬) is shown in red, and the various superconducting materials are indicated as •. The blue circle gives the position of all (*RE*)BCO superconductors with optimum oxygen content. SOFFA is the abbreviation for SmO${}_{1-\delta }$F${}_{\delta }$FeAs, the IBS superconductor with the highest $T_{c}$, GOFFA, and LOFFA are Gd/LaO${}_{1-\Delta }$F${}_{\Delta }$FeAs and LSCO is the abbreviation for (La, Sr)CuO${}_{4}$. NCCO stands for Nd${}_{2}$CeCuO${}_{4}$, which is an n-type superconductor. Tl-1201, Tl-1212, and Tl-1234 are members of the Tl-based HTSc family with equivalent Cu-O-planes, and Bi-2201, Bi-2212, and Bi-2223 are members of the Bi-based HTSc family. The inset presents the data calculated here (see also Table 1) in more detail.

Figure 7 presents the Roeser–Huber line (drawn in red) and the data obtained for the various *RE*BCO-compounds calculated here (inset). The red line follows the equation of a particle in a box with the energy $h^{2}/\left(8M_{L}x^{2}\right)=\pi k_{B}T_{c}$ for n=1. The theoretical slope is $m=h^{2}/\left(2\pi k_{B}M_{L}\right)=$ 3.02586 × 10${}^{-18}$ m${}^{2}$ K. The datapoints (•) showing the various superconducting materials stem from the present work and Refs. [9,10,11,12,13,68]. The calculations performed cover p-type, as well as n-type superconductors, copper-based ones, as well as several IBS materials. It is obvious that a large variety of HTSc materials, as well as metallic superconductors (elements and alloys) fulfill the Roeser–Huber condition. The correlation for HTSc is very good, whereas in the case of the metallic superconductors some deviations prevail, but only within a narrow error margin of 1.23%, which is remarkably small. This clearly illustrates the importance of this relation for the superconducting materials, and it offers an unique approach to calculate (or verify) $T_{c}$ of superconductors making use of available crystallographic information.

### 3.4. Effect of *LRE*-Ba-Solid Solution and the Spatial Variation of $T_{c}$

Due to the solid solution of the form *LRE*${}_{1+z}$Ba${}_{2-z}$Cu${}_{3}$O${}_{7-\delta }$ between the light-rare earth elements (LRE, i.e., Nd, Eu, Gd, Sm—marked red in Figure 7) and Ba [55,69,70,71], a formation of *LRE*-rich unit cells, where the LRE-ion can replace the Ba-ion of similar size. The LRE-rich material has a considerably lower $T_{c}$ as compared to the “normal” unit cell of *LRE*-BCO. The Nd123ss solid solution prevails in a relatively large region, 0.04 ≤z≤ 0.6, which is due to the similar ionic radii of Ba${}^{2+}$ and the light rare earth ions *LRE*${}^{3+}$. To obtain optimally superconducting samples, a preparation in a controlled oxygen atmosphere of 1% O${}_{2}$ in Ar is required [70], even though there are several reports of fabricating, e.g., GdBCO in air [72]. All these materials do show a pronounced fishtail character in the magnetization loops, which is even more pronounced in the ternary compound (Nd${}_{0.33}$Eu${}_{0.33}$Gd${}_{0.33}$)BCO (abbreviated NEG), as described in Ref. [73]. This solid solution is also the reason for the difficulties reported in [38] to obtain good quality NdBCO thin films with high $T_{c}$ using the standard conditions as for YBCO.

According to Ref. [70], forcing the excess Nd to order on the Ba-sites, the oxygen disorder on the chain site might be reduced, and, thus, the resulting $T_{c}$ of this compound may be increased. The Nd${}^{3+}$-ion has the closest ionic radius to Ba${}^{2+}$. On the other hand, the charge transfer to the Cu-O-plane is altered when replacing Ba${}^{2+}$ with Nd${}^{3+}$. In Ref. [69], the diagram of $T_{c}$ as a function of *z* (fully oxygenated samples) reveals the existence of two plateaus at z=0...0.08 and at z=0.25...0.3. The first plateau at low *z* is much larger as in Figure 4, and the second plateau is much less pronounced as in the case of YBCO. Of course, we must note here that all samples were fully oxygen-loaded, and the variation of $T_{c}$ is due to the variation of the Nd123ss solid solution.

To calculate $T_{c}$ of the Nd-rich compound, it is assumed that replacing Ba by Nd leads to an additional doping effect to the superconducting Cu-O-plane. Thus, we have a double doping by Nd and the oxygen content in this system. To describe this double-doping effect, Roeser et al. [68] were calculating the doping distances for both contributions separately, and then put the two doping patterns together like a Moiré effect. The resulting characteristic distance, *x*, describing the $T_{c}$ value of such a compound is then much larger than those of the two individual contributions. Using the procedure described in Ref. [68], the $T_{c}$ of the Nd-rich compound can be calculated to be 65 K for optimum oxygen doping (δ= 0.04 and z= 0.3). More details of this calculation will be presented in a subsequent paper [74].

### 3.5. Strain-Controlled $T_{c}$ in Coated Conductors and High Pressure Effects

Awaji et al. [37] have succeeded to prepare untwinned *RE*-123 coated conductor samples. This enabled them to discuss the effect of strain from the substrate on $T_{c}$ of the samples. This observation is strongly related to the effects of high pressure on the YBCO unit cell, as discussed by Welp et al. [75] and Veal et al. [76].

**Figure 8:**
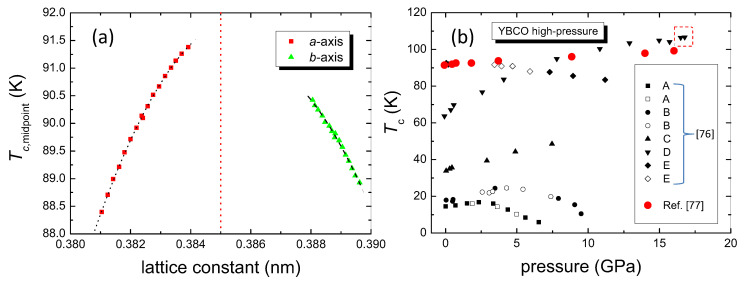
(**a**) Relationship between $T_{c}$ and the lattice constants at room temperature, redrawn from Ref. [37]. The dashed red line indicates the optimum condition for the highest $T_{c}$ with a=b= 0.385 nm. The dotted and dashed lines are polynomal fits to the data. (**b**) shows the effect of applying pressure to various YBCO crystals (experimental runs A–E, see main text, taken from Ref. [76]). Closed symbols are for increasing pressure, open symbols are for decreasing pressure. The red bullets (•) indicate data from Ref. [77]. The record $T_{c}$ obtained in the YBCO system was found to be 106.7 K (indicated by a dashed-red box) at 17 GPa.

In their paper, Awaji et al. show that the strain effects on the *a*- and *b*-axis have opposite signs (see Figure 8a, thus the strain effects on the *a*- and *b*-axes are cancelling out each other). From their data, it is obvious that the maximum $T_{c}$ in YBCO is obtained when a=b= 0.385 nm, as indicated by the red dashed line. Therefore, we also calculated $T_{c}$ of YBCO using this value (see Table 1 above). If we use the data for *a*, as shown in Figure 8a, and a corresponding *b* calculated via orthorhombicity = a/b, we can nicely reproduce the respective measured transition temperatures using the Roeser–Huber formula. This again is a proof of the underlying concept of the Roeser–Huber approach.

The application of high pressure to YBCO crystals shows several different features as illustrated in Figure 8b. For optimally doped YBCO, there is only a small increase in $T_{c}$ with pressure, as found by several authors [76,77]. This situation changes when samples with higher oxygen deficiency are analyzed. Figure 8b gives the data of Sadewasser et al. [76] as an example. Samples A, B, and C had a $T_{c}$ lower than the plateau value of 57 K. Sample D had an ambient pressure $T_{c}$ of 62 K, and sample E was optimally doped. Thus sample D, consisting of a mixture of ortho-I and ortho-II clusters, showed the largest increase with pressure of all samples studied and is at high pressures similar to the data of the crystal studied by Wijngaarden et al. [77]. Regarding the ASYNNNI model outcome and the simulations of Poulsen [35], it is straightforward to conclude that application of pressure forces an ordering of the existing oxygen atoms in the Cu-O-plane. Then, there is always an optimum configuration which gives the highest $T_{c}$, and further application of pressure causes again a reduction in $T_{c}$. Thus, the $T_{c}$ of YBCO cannot be increased to higher values than this optimum configuration. To obtain an exact result of $T_{c}$ under pressure, one must consider also the behavior of the *c*-axis, and as it was discussed recently, also the vacancies of the apical oxygen [78,79,80]. It is, of course, a pity that practically all experimental data for YBCO available in the literature do not give data for the crystallographic axes or the respective volume of the unit cell, so we cannot use the Roeser–Huber formalism to calculate $T_{c}$ under applied pressure, but we can determine the underlying length *x* from the measured $T_{c}$-values (see the right axis of Figure 5). So, *x* for the record $T_{c}$ of 106.7 K (dashed-red box in Figure 8b) turns out to be 2.551 nm (a= 0.3644 nm), which implies a small compression of the ortho-I clusters.

## 4. Conclusions

To conclude, we have revisited the calculation of $T_{c}$ of YBCO using the Roeser–Huber formula. Using the literature on oxygen ordering, we can now explain the two different Cu-O-Cu-O-chains required for the calculation of nearly stoichiometric (δ= 0.04) and oxygen-deficient YBCO (δ= 0.45). Then, the Roeser–Huber calculations were applied to various *RE*BCO compounds, where the calculated $T_{c}$’s fit the data of bulk superconducting samples quite well. Furthermore, we could discuss the effect of the Nd-Ba-solid solution in the NdBa${}_{2}$Cu${}_{3}$O${}_{y}$-system, and the influence of strain on $T_{c}$, as well as the pressure dependence of YBCO. Furthermore, the characteristic length *x*, which describes the oxygen-doping distance, gives important information on the size of the $\delta T_{c}$-pinning sites, which are responsible for the fishtail effect observed in magnetization measurements of (*RE*)BCO samples.

## Figures and Tables

**Table 1:** The distance *x*, ${\left(2x\right)}^{2}$, the calculated energies, $\Delta_{\left(0\right)}$, and the calculated $T_{c}$ using the *a*-axis (data of Figure 5) for the various *RE*BCO materials. The data for YBCO in the first two rows are the data from Ref. [9] with a=b, and the next two rows use a= 0.385 nm as the optimum found in [37]. All $T_{c}$-data correspond well to the data obtained on bulk samples; in the case of thin films as discussed in Ref. [38], the correlation is not so straightforward. NdBCO-SS is discussed in Section 3.4 below.

Material	Lattice Parameter	*x* (10${}^{-9}$ m)	(2*x*)${}^{2}$ (10${}^{-17}$ m${}^{2}$)	$\Delta_{\left(0\right)}$ (meV)	$T_{c}$ ((K))
YBCO (δ= 0.04)	a=b= 0.39	2.73	2.981	25.2	93
YBCO (δ= 0.45)		3.49	4.872	15.4	57
YBCO (δ= 0.04)	a=b= 0.385	2.695	2.905	25.9	95.6
YBCO (δ= 0.45)		3.444	4.744	15.9	58.55
*RE*BCO (δ= 0.04)	a=b				
NdBCO	3.9128	2.7390	3.0008	25.06	92.57
SmBCO	3.904	2.7328	2.9873	25.18	92.99
EuBCO	3.8992	2.7294	2.9799	25.24	93.22
GdBCO	3.8968	2.7278	2.9763	25.27	93.34
DyBCO	3.8887	2.7209	2.9613	25.4	93.81
YBCO	3.8863	2.7204	2.9603	25.41	93.84
HoBCO	3.8846	2.7192	2.9577	25.43	93.92
ErBCO	3.8809	2.7166	2.9520	25.48	94.10
TmBCO	3.8758	2.7131	2.9443	25.5	94.35
YbBCO	3.8711	2.7098	2.9371	25.6	94.58
NdBCO SS	av. (*a,b*) 3.8885	3.4780	4.8385	15.56	57.41
	z= 0.3				

## Data Availability

The data presented in this study are available on request from the corresponding author.
